# *Classification in Psychiatry*: Does it deliver in schizophrenia and depression?

**DOI:** 10.1186/1752-4458-1-7

**Published:** 2007-12-03

**Authors:** Graham W Mellsop, David B Menkes, Selim M El-Badri

**Affiliations:** 1 Waikato Clinical School, University of Auckland, New Zealand

## Abstract

**Background:**

In the context of ongoing work to develop the next iteration of psychiatric classification systems, we briefly review the performance of current systems against their own stated objectives, for two major diagnostic groupings.

**Discussion:**

In the major groupings of schizophrenia and depression, experience over the last 50 years has highlighted particular inadequacies in the utility and validity of available classifications.

**Summary:**

Advances in psychiatric knowledge and practice notwithstanding, present classification systems would be enhanced by the incorporation of dimensional components. Minor tinkering with current systems will reflect only a missed opportunity. Improving classification will facilitate quality improvement of mental health systems.

## Background

As a specialised domain within health sciences, psychiatric diagnostic practice has evolved under the influence of the biomedical model, stimulated in part by the success of rigorous and useful models of scientific classification, such as the periodic table and Linnaean biological taxonomy[1].

Systematic classifications in psychiatry were first recognisably developed by Pinel, but it is usually accepted that we owe most to the work of Kraepelin and Bleuler[1]. The labels available for mental disorders have evolved from those baselines, with periodic, formal re-evaluation under the guidance of the World Health Organisation, as new editions of the International Classification of Diseases (ICD) have been developed [2]. Also widely used is the 4^th ^Edition of the Diagnostic and Statistical Manual (DSM-IV) of the American Psychiatric Association[3].

In general, psychiatric literature, service organisation and funding, clinicians and researchers have worked within this framework. Nonetheless, there has always been a trickle of thoughtful dissenters and research based questions raised, sometimes about the fundamental approach, but more often about the detail [4-9].

Chapter V of ICD-10, the Classification of Mental or Behavioural Disorders[2] "...is intended for general clinical, educational and service use." The Text Revision of DSM-IV[3] is more specific "our highest priority has been to provide a helpful guide to clinical practice," amplifying this to include: -

• A means by which the profession *communicates *briefly and clearly within itself about clinically recognisable conditions for which it has professional responsibility for diagnosis, care, or research.

• When possible, a useful *guide to current treatments*.

• Information about the *likely outcome *of psychiatric disorders with and without treatment.

• What is known of aetiology or *pathophysiological *processes.

The specificity apparent in the DSM guiding principles accords with the oft quoted views of Robins and Guze[10] on comprehensive criteria for evaluating the validity of a psychiatric disorder. Such demanding criteria are far from being realized for many psychiatric disorders [11]. Recent work in Australasia, South America and East Asia suggests that practising psychiatrists have more limited views, or expectations, on the purpose of psychiatric classification [12]. It remains an open question whether the Robins and Guze criteria need to be applied and implemented more effectively, or rethought altogether.

From the above, we conclude that psychiatric classificatory systems are intended to facilitate communication of information regarding:

• Description of disorders in order to promote understanding by colleagues, patients, and others

• Pathogenesis

• Indicated treatment choices

• Prognosis

• Categories useful for epidemiology, outcomes and other research

Below, we consider schizophrenia and depression, the two groups of diagnoses most commonly used in psychiatry. For each diagnostic group we note key published perspectives on the performance of current classification against the above objectives.

### Schizophrenia

Current classificatory descriptions of schizophrenia are detailed, precise and specific, and as such allow reliable communication regarding how the disorder 'feels' to those affected, and 'looks' to clinicians and family. Classifications achieved an acceptable degree of inter-rater reliability in field trials[2,3] but perform far less reliably in standard clinical practice[11]. The 37 four-digit codes available under the ICD-10 general category of schizophrenia provide a degree of detail likely to be irrelevant in many countries [13].

There is little in either ICD or DSM regarding aetiology, an important shortcoming in light of evidence that both genetics and environment, separately and in combination, confer risk for this disorder in various populations [14,15]. There is also evidence of  physiological, anatomical and immunological abnormalities in  schizophrenia that strongly suggest one or more brain pathologies [16,17]. It follows that improved diagnostic validity will, *inter alia*, magnify the signal-to-noise ratio for detecting causal mechanisms operating in schizophrenia, particularly if there exist distinct biological or other pathogenic subgroups. Equally, better mechanistic understanding of schizophrenia will help to validate diagnostic subtypes, and their treatment implications.

A key problem, evident since the time of Bleuler, has been that the prognosis in 'schizophrenia' has appeared variable, from complete recovery to progressive dysfunction and invalidism[18]. This problem prompted the delineation of 'schizophreniform disorder' in DSM, phenomenologically identical to acute schizophrenia but with a brief time course, more frequent remission, and better prognosis. Similarly, the work of Robins and Guze[10] indicates that what has been called 'mild' or good prognosis schizophrenia is fundamentally different from typical or 'process' schizophrenia and it may thus be a misnomer to refer to the former as schizophrenia at all. Such clinical reasoning, bolstered by carefully collected outcome data and accumulating evidence of pathogenic mechanisms, indicates that a categorical definition of schizophrenia is likely to remain valid, provided that similar disorders can be reliably distinguished. A supplementary dimensional description of phase and severity holds promise as a guide to management and prognosis[19].

Treatment guidelines for schizophrenia include a variety of psychosocial interventions, but centre around antipsychotic drugs[18,20]. Other available psychotropics, including mood stabilisers, antidepressants and hypnosedatives, also have an apparent role, raising serious questions about any specificity between diagnosis and indicated medication. Moreover, clinical response to individual or grouped agents is unpredictable, and treatment decisions are influenced by many patient variables other than diagnosis [18,20]. For example, treatment needs in chronic psychosis are more powerfully predicted by symptom intensity than by diagnosis *per se *[9].

### Depression

Widespread debate about the validity of contemporary classification of depression stems from the fact that the therapeutic and prognostic implications of mood disorder diagnosis have repeatedly proved to be unreliable. Thus Parker [21] has argued convincingly that the concept of major depression suffers from problems of both reliability and validity, and crucially "fails the test of providing meaningful information about aetiology, prognosis and treatment". One perennial difficulty is establishing a valid diagnostic threshold of symptom severity or functional impairment – particularly problematic since everyone experiences fluctuating depressive symptoms to some extent. It is not surprising therefore that the use of the diagnostic label 'depressive disorder' has created a spurious impression of understanding [22,23]. It does not tell us why and how a person became depressed nor does it indicate degree of disability, duration, or the risk to self or others. Similarly, it does not inform us about what specific treatment (e.g. ECT, antidepressant medication or psychotherapy) would be appropriate or helpful. Furthermore, there is a disparity between the descriptive diagnostic labels, the real life situation, and day-to-day clinical practice. This apparent lack of correspondence between diagnosis and disorder is partly related to the failure of contemporary classification systems to recognise and address the inherent boundary ambiguities in depressive disorder[23]. The usefulness of diagnostic categories for epidemiology, outcomes and other research in mood disorders is likely to continue, but serious concerns regarding validity call for consideration of dimensional diagnostic alternatives [24].

## Conclusion

This report highlights the degree to which the performance of ICD-10 and DSM-IV fall short of their own objectives. It is time to acknowledge that the 'classification emperor' is less well-clothed than our daily practice requires [25]. Existing categories also constrain thinking about how to measure and address unmet need in populations. New, more valid, means of classification need to be developed, perhaps incorporating concepts derived from non-categorical thinking. Dimensional classification may, for example, usefully inform the description of unipolar depression, anxiety and personality disorders [24,26]. A "quantitative" axis incorporating such dimensions and other guides to treatment and progress, such as disorder staging and substance use, is a classificatory evolution whose time has come. For these disorders at least, dimensional description offers potential advances in the prediction of both treatment choice and longitudinal course, while retaining improvements in inter-clinician communication developed over the last 25 years. With regard to the major psychoses, available evidence is more supportive of the validity of categorical diagnoses but suggests these need to be supplemented with dimensional or illness-phase classification. Simplification of current, overly  complex subcategories is clearly warranted; dimensional measures retain complementary importance as predictors of impairment and prognosis.

## Summary

Official systems of psychiatric classification have been evolving for more than 100 years and underpin the organisation of services, individual clinicians' work, and most psychiatric research. Sporadic reviews have challenged the validity and usefulness of some of the key categories in ICD and DSM. This article reflects on classification system performances against their own claimed objectives, in two diagnostic areas. We conclude that evident inadequacies can no longer be ignored and that dimensional approaches to classification offer one way forward.

## List of abbreviations used

DSM: Diagnostic and Statistical Manual of Mental Disorders;

ICD: International Classification of Diseases.

## Competing interests

The author(s) declare that they have no competing interests.

## Authors' contributions

All three authors contributed significantly to the background literature review, to the development of the argument, and to drafting the text.

## References

[B1] Pichot P (1986). Nosological Developments in European Psychiatry and Psychopharmacology. Pharmacopsychiatry.

[B2] (2002). The ICD10 Classification of Mental Behavioural Disorders: Clinical Description and Diagnostic Guidelines.

[B3] (2000). Diagnostic and Statistical Manual of Mental Disorders 4^th ^Ed, Text Revision DSM-IV-TR.

[B4] Widiger T (2005). Five factor model of personality disorder: Integrating science and practice. Journal of Research in Personality.

[B5] Jablensky A (2005). Categories, dimensions and prototypes: critical issues for psychiatric classification. Psychopathology.

[B6] Goldberg DP (1996). A dimensional model for common mental disorders. The British Journal of Psychiatry.

[B7] Helzer JE, Kraemer HC, Krueger RF (2006). The feasibility and need for dimensional psychiatric diagnoses. Psychological Medicine.

[B8] Boteva K, Lieberman J (2003). Reconsidering the Classification of Schizophrenia and Manic Depressive Illness – A Critical Analysis and New Conceptual Model. World Journal of Biological Psychiatry.

[B9] McGorry PD, Hickie IB, Yung AR, Pantelis C, Jackson HJ (2006). Clinical Staging of psychiatric disorders: A heuristic framework for choosing earlier, safer and more effective interventions. Australian and New Zealand Journal of Psychiatry.

[B10] Robins E, Guze SB (1970). Establishment of Diagnostic Validity in Psychiatric Illness: Its Application to Schizophrenia. American Journal of Psychiatry.

[B11] Baca-Garcia E, Perez-Rodriquez MM, Basurte-Villamor I, Fernandez del Moral AL, Jimenez-Arriero MA, Gonzalez de Rivera JL, Saiz J, Oquendo MA (2007). Diagnostic stability of disorders in clinical practice: A prospective cohort study. British Journal of Psychiatry.

[B12] Mellsop GW, Banzato CEM, Shinfuku N, Nagamine M, Pereira MEC, Dutu G (2007). An International study of the views of psychiatrists on present and preferred characteristics of classifications of psychiatric disorders. International Journal of Mental Health.

[B13] Müssigbrodt H, Michels R, Malchow CP, Dilling H, Munk-Jørgensen P, Bertelsen A (2000). Use of the ICD-10 classification in psychiatry: An international survey. Psychopathology.

[B14] Fearon P, Morgan C (2006). Environmental Factors in Schizophrenia: The Role of Migrant Studies. Schizophrenia Bulletin.

[B15] Ciompi L (1989). The dynamics of complex biological psychosocial systems. Four fundamental psycho-biological mediators in the long-term evolution of schizophrenia. Br J Psychiatry Suppl.

[B16] Knight JG, Menkes DB, Highton J, Adams DD (2007). Rationale for a trial of immunosuppressive therapy in acute schizophrenia. Molecular Psychiatry.

[B17] Haroutunian V, Davis KL (2007). Introduction to the Special Section: Myelin and oligodendrocyte abnormalities in schizophrenia. International Journal of Neuropsychopharmacology.

[B18] Byrne P (2007). Managing the Acute Psychotic Episode. BMJ.

[B19] Van Os J, Gilvarry C, Bale R, Van Horn E, Tattan T, White I, Murray R, on behalf of the UK700 Group (1999). A comparison of the utility of dimensional and categorical representations of psychosis. Psychological Medicine.

[B20] (2003). National Collaborating Centre for Mental Health Schizophrenia: Full National Clinical Guideline on Core Interventions in Primary and Secondary Care.

[B21] Parker G (2005). Beyond major Depression. Psychological Medicine.

[B22] Farmer A, McGuffin P (1989). The classification of the depressions: Contemporary confusion revisited. British Journal of Psychiatry.

[B23] Kendler KS, Gardner CO (1998). Boundaries of Major Depression: An evaluation of DSM-IV criteria. American Journal of Psychiatry.

[B24] Slade T, Watson D (2006). The Structure of Common DSM 4 and ICD 10 Mental Disorders in the Australian General Population. Psychol Med.

[B25] Mellsop GW, Menkes DB, El-Badri S (2007). Releasing Psychiatry from the Constraints of Categorical Diagnosis. Australasian Journal of Psychiatry.

[B26] Krueger RF, McGrue M, Iacono WG (2001). The Higher Order Structure of Common DSM Mental Disorders: Internalisation, Externalisation, and their Connections to Personality. Personality and Individual Differences.

